# Patients’ treatment limitations as predictive factor for mortality in COVID-19: results from hospitalized patients of a hotspot region for SARS-CoV-2 infections

**DOI:** 10.1186/s12931-021-01756-2

**Published:** 2021-06-04

**Authors:** Stephan Budweiser, Şevki Baş, Rudolf A. Jörres, Sebastian Engelhardt, Stefan von Delius, Katharina Lenherr, Jens Deerberg-Wittram, Andreas Bauer

**Affiliations:** 1 Department of Internal Medicine III, Division of Pulmonary and Respiratory Medicine, RoMed Hospital Rosenheim, Pettenkoferstrasse 10, 83022 Rosenheim, Germany; 2 Institute and Outpatient Clinic for Occupational, Social and Environmental Medicine, Ludwig-Maximilians-University, Munich, Germany; 3 Department of Emergency, RoMed Hospital Rosenheim, Rosenheim, Germany; 4 Department of Internal Medicine II, RoMed Hospital Rosenheim, Rosenheim, Germany; 5 Internal Intensive Care Medicine Unit, RoMed Hospital Rosenheim, Rosenheim, Germany; 6 RoMed, Rosenheim, Germany; 7 Institute for Anesthesiology and Surgical Intensive Care Medicine, RoMed Hospital Rosenheim, Rosenheim, Germany

**Keywords:** COVID-19, Mortality, Prognostic factors, Life supporting care limitation

## Abstract

**Background:**

In hospitalized patients with SARS-CoV-2 infection, outcomes markedly differ between locations, regions and countries. One possible cause for these variations in outcomes could be differences in patient treatment limitations (PTL) in different locations. We thus studied their role as predictor for mortality in a population of hospitalized patients with COVID-19.

**Methods:**

In a region with high incidence of SARS-CoV-2 infection, adult hospitalized patients with PCR-confirmed SARS-CoV-2 infection were prospectively registered and characterized regarding sex, age, vital signs, symptoms, comorbidities (including Charlson comorbidity index (CCI)), transcutaneous pulse oximetry (SpO_2_) and laboratory values upon admission, as well as ICU-stay including respiratory support, discharge, transfer to another hospital and death. PTL assessed by routine clinical procedures comprised the acceptance of ICU-therapy, orotracheal intubation and/or cardiopulmonary resuscitation.

**Results:**

Among 526 patients included (median [quartiles] age 73 [57; 82] years, 47% female), 226 (43%) had at least one treatment limitation. Each limitation was associated with age, dementia and eGFR (p < 0.05 each), that regarding resuscitation additionally with Charlson comorbidity index (CCI) and cardiac disease. Overall mortality was 27% and lower (p < 0.001) in patients without treatment limitation (12%) compared to those with any limitation (47%). In univariate analyses, age and comorbidities (diabetes, cardiac, cerebrovascular, renal, hepatic, malignant disease, dementia), SpO_2_, hemoglobin, leucocyte numbers, estimated glomerular filtration rate (eGFR), C-reactive protein (CRP), Interleukin-6 and LDH were predictive for death (p < 0.05 each). In multivariate analyses, the presence of any treatment limitation was an independent predictor of death (OR 4.34, 95%-CI 2.10–12.30; p = 0.001), in addition to CCI, eGFR < 55 ml/min, neutrophil number > 5 G/l, CRP > 7 mg/l and SpO_2_ < 93% (p < 0.05 each).

**Conclusion:**

In hospitalized patients with SARS-CoV-2, the percentage of patients with treatment limitations was high. PTL were linked to age, comorbidities and eGFR assessed upon admission and strong, independent risk factors for mortality. These findings might be useful for further understanding of COVID-19 mortality and its regional variations.

*Clinical trial registration* ClinicalTrials.gov Identifier: NCT04344171

**Supplementary Information:**

The online version contains supplementary material available at 10.1186/s12931-021-01756-2.

## Background

The SARS-CoV-2 pandemic is one of the biggest challenges for the global health-care system involving economic, medical and ethical dimensions. Since the first outbreak in Wuhan, China [1], this pandemic has spread over almost all regions of the word, reaching currently more than 38.7 million global cases with 1,094,979 deaths [2].

In Europe, the region of Lombardy, northern Spain, eastern France and the United Kingdom experienced high incidence rates of SARS-CoV-2 infections during the first wave, showing in-hospital mortality rates from 17 to 39% [3–6]. Especially in the region of Lombardy, deficits in health-care resources became overt but systematic analyses of this chapter of COVID-19 management are currently not available. In Germany, according to the Robert-Koch-Institute [7], 341,223 SARS-CoV-2 infections have been counted until October 15, 2020, while shortages of health-care resources have not been reported. Nevertheless, some regions, such as the region of Rosenheim, developed into hotspots, with 2806 cases until June 30, 2020, i.e. about 120 per 100,000 inhabitants.

In order to allocate health-care resources in the best manner and make evidence-based decisions, knowledge of predictors of the clinical course of COVID-19 is required. Age, sex, comorbidities, virus load and biomarkers have been identified as prognostic factors on the patients’ side [8–16]. From the health-care system perspective, the availability of critical resources such as beds in intensive care units (ICU), the scope of medical specialization and skills, personnel resources, and technical equipment could affect the outcome. Given the severity of the disease, patients’ treatment limitations (PTL), including “do not intubate (DNI)”/”do not resuscitate (DNR)” orders might also be important prognostic factors but appear to have been ignored until now [17]. It may be difficult to address this question in large multicenter studies involving different hospitals with their individual processes. In contrast, a real-life analysis of a rather homogeneous population, with no limitations in medical capacity and detailed knowledge of patients’ characteristics may be better suited to address the relevance of individual treatment limitations for mortality. Thus, the present study analyzed mortality from COVID-19 and including known risk factors in patients hospitalized with SARS-CoV-2 focusing on the role of treatment limitations.

## Methods

### Study population and assessments

The RoMed Health System comprising four hospitals (Rosenheim, Bad Aibling, Wasserburg, Prien a. Chiemsee) is a major health-care provider in southeast Bavaria, Germany, to a population of 350,000 people. All adult patients hospitalized between March 1 and June 30, 2020, with SARS-CoV-2 infection confirmed by reverse transcription-polymerase chain reaction (RT-PCR) from oro- and/or nasopharyngeal swabs, sputum or bronchoalveolar lavage were included into the analysis. Further details can be found in Additional file 1.

For patients’ description, we used sex, age, and data on symptoms, smoking status, body temperature, heart rate, blood pressure, transcutaneous pulse oximetry (SpO_2_) and laboratory values (blood cell counts, LDH, ALT, GGT, creatinine, CRP, Interleukin-6) upon admission. Estimated glomerular filtration rate (eGFR) was calculated using the Chronic Kidney Disease-Epidemiology Collaboration (CKD-EPI) equation [18]. Additional assessments comprised the date of admission, presence of comorbidities (from medical records) including the Charlson comorbidity index (CCI) (without age if this was considered separately) [19].

Moreover, we assessed DNR/DNI referring to orotracheal intubation and cardiopulmonary resuscitation, additionally the willingness to accept ICU-therapy in general; this was collectively termed as “Patients’ Treatment Limitations” (PTL). These decisions were regularly based on informed consent between patients and/or their relatives and the treating physicians taking into account patients’ personal preferences, comorbidities and age. The result was documented in the patients’ files from which it was retrieved.

### Course and follow-up

We recorded admission to ICU, type of respiratory support, length of mechanical ventilation (MV) and ICU stay, date of death within or discharge from a RoMed Hospital, or transfer to another hospital and death or discharge regarding that hospital. All patients were followed until discharge or death.

### Statistical analysis

Due to deviations from normal distribution in a number of variables according to the Shapiro–Wilk-test and Kolmogorov–Smirnov-test, data is presented as median and quartiles. The Mann–Whitney-U-test was employed for comparisons between groups for continuous variables, and Fisher’s exact test for categorical variables. To account for skewed data distributions of laboratory parameters without introducing complex transformations, and to facilitate the clinical interpretation, we determined their Receiver Operating Characteristics (ROC) and identified the best cut-off values, using the Youden criterion. The target variable was death. All laboratory parameters were subsequently used as binary categories. Moreover, the CCI was set to a maximum value of 10, in order to avoid statistically unfavourable effects from three extreme values on the confidence intervals; this did not affect the pattern of statistical significance.

To analyze single predictors, we used univariate regression analysis and contingency tables, and to identify independent predictors of mortality or treatment limitations, multiple logistic regression analysis, whereby statistical significance and confidence intervals were determined via the bootstrap approach using 1000 samples. In the logistic regression analyses it was assessed to which extent death or the three single PTL or their combinations were linked to the predictors age, sex, peripheral artery disease, cardiovascular disease, obstructive airway disease (asthma and/or COPD), malignant disease, dementia, CCI, and the binary categories regarding eGFR, neutrophil number, CRP and SpO_2_ (see below). In case of mortality, the analysis was repeated by adding treatment limitations, either separately or in form of combinations, in order to determine their differential role. Due to their high correlation, they were not included simultaneously. To keep the statistical power high, the multivariate analyses included only variables with the highest F-value among those that turned out to be highly correlated. We also limited the number of laboratory parameters to four to avoid problems from collinearity and a reduction of power when using a multitude of predictors. Using this approach, parsimonious final sets of predictors were obtained. p-values < 0.05 were considered statistically significant. For analysis, the statistical software SPSS (Version 25.0 and 26.0; Chicago, IL, USA) was used.

## Results

### Study population

The total population comprised 245 (46.6%) female and 281 (53.4%) male patients with an age of 73.0 [57.0; 82.0] years (Table 1). Patients presented with normal blood pressure, but slightly elevated body temperature, elevated heart rate and hypoxemia despite hyperventilation (Table 1). The most frequent symptoms were fever (52.9%), dyspnea (51%) and cough (44.7%) (Table 2). Systemic hypertension (50.6%), left-heart failure (29.7%) and renal disease (27.1%) were the most frequent comorbidities (Table 2). A significant cardiovascular disease (defined as coronary heart disease, or left heart failure, or atrial fibrillation) occurred in 40.9% of patients. The CCI without age was 2 (0; 4), the score including age 2 (5; 7). 57 patients (10.8%) had no significant comorbidities. Further information regarding the clinical management and course of the disease is provided in Additional file 1.

**Table 1:** Patients’ characteristics including comparison of patients with or without any treatment limitation (PTL)

Variable	All patients (n = 526)		No limitation (n = 300)		Any limitation (n = 226)		p-value
	n	Median (quartiles)	n	Median (quartiles)	n	Median (quartiles)	
Demographics/vital parameters							
Sex (%)	526	♂ 53.4; ♀ 46.6	300	♂ 55.7; ♀44.3	226	♂ 50.4; ♀ 49.6	0.252
Age (years)	526	73 (57; 82)	300	60 (51; 73)	226	81 (76; 87)	**< 0.001**
Pulse rate (1/min)	523	84 (76; 95)	299	84 (78; 97)	224	82 (73.3; 94)	0.065
sBP (mmHg)	523	129 (113; 140)	299	130 (115; 140)	224	126 (110; 140)	0.162
dBP (mmHg)	523	75 (65; 84)	299	77 (70; 85)	224	70 (60; 80)	**< 0.001**
Body temperature (°C)	525	37.6 (36.8; 38.4)	300	37.7 (37; 38.5)	225	37.4 (36.7; 38.2)	**0.007**
SpO_2_ (%)	506	94 (91; 96)	285	94 (92; 96.3)	220	93 (90; 96)	**0.002**
Laboratory parameters upon admission							
Hemoglobin (g/dl)	524	13.3 (11.8; 14.6)	300	13.5 (12.2; 14.8)	224	12.9 (11.3; 14.2)	**< 0.001**
Hematocrit (%)	524	38.6 (34.5; 42.0)	300	39.4 (35.3; 42.3)	224	37.9 (33.3; 41.7)	**0.007**
Thrombocytes (G/l)	524	197.5 (150.0; 252.0)	300	198.5 (152.0; 249.8)	224	190 (148.3; 255.8)	0.885
Leucocytes (G/l)	524	6.5 (4.7; 8.9)	300	6.1 (4.6; 8.4)	224	6.9 (5.0; 9.8)	**0.001**
Lymphocytes (G/l)	456	0.9 (0.7; 1.3)	262	1 (0.7; 1.4)	194	0.9 (0.6; 1.3)	**0.014**
Neutrophils (G/l)	456	5.1 (3.4; 7.6)	262	4.7 (3.2; 6.8)	194	5.4 (3.8; 8.5)	**0.001**
Creatinine (µmol/l)	517	1.1 (0.9; 1.5)	294	1 (0.8; 1.3)	223	1.4 (1.0; 2.0)	**< 0.001**
eGFR (ml/min)	517	61.1 (37.9; 82.2)	294	73.6 (55.2; 88.1)	223	42.8 (26.3; 62.2)	**< 0.001**
ALT (U/l)	473	25.0 (17.4; 39.0)	271	28 (19.6; 43.1)	202	21.9 (15.2; 32.5)	**< 0.001**
GGT (U/l)	448	41.0 (22.3; 79.0)	251	41 (23.0; 75.0)	197	41 (22.0; 92.5)	0.591
LDH (U/l)	419	311.0 (231.0; 402.0)	238	309 (229.8; 394.8)	181	315 (235.0; 402.5)	0.674
CRP (mg/dl)	519	6.0 (2.3; 11.5)	297	5.4 (1.9; 10.2)	222	6.5 (3.1; 12.1)	**0.014**
IL-6 (pg/ml)	91	66.5 (27.4; 127.0)	55	73.3 (27.7; 127.0)	36	63.6 (24.8; 128.8)	0.858

Bold p-values represent statistical significance (< 0.05)

*sBP* systolic blood pressure, *dBP* diastolic blood pressure, *SpO*_*2*_ pulse oxygen saturation, *eGFR* estimated glomerular filtration rate, *ALT* alanine aminotransferase, *GGT* gamma-glutamyl transferase, *LDH* lactate dehydrogenase, *CRP* C-reactive protein, *IL-6* Interleukin-6

**Table 2:** Patients’ characteristics including comparison of patients with or without any treatment limitation (PTL)

Variable	All patients (n = 526)		No limitation (n = 300)		Any limitation (n = 226)		p-value
	n	%	n	%	n	%	
Symptoms							
Cough	235	44.7	161	53.7	74	32.7	**< 0.001**
Sputum	33	6.3	18	6.0	15	6.6	0.856
Sore throat	13	2.5	12	4.0	1	0.4	**0.009**
Fever/chills	278	52.9	169	56.3	109	48.2	0.078
Diarrhoea	73	13.9	50	16.7	23	10.2	**0.041**
Nausea	28	5.3	15	5.0	13	5.8	0.700
Loss of appetite	70	13.3	31	10.3	39	17.3	**0.027**
Fatigue	179	34	100	33.2	79	35.0	0.711
Dyspnea	268	51	158	52.7	110	48.7	0.379
Headache	58	11	52	17.3	6	2.7	**< 0.001**
Loss of smell/taste	38	7.2	30	10.0	8	3.5	**0.006**
Comorbidity							
Systemic hypertension	266	50.6	117	39.0	149	65.9	**< 0.001**
Diabetes mellitus	122	23.2	54	18	68	30.1	**0.002**
Left-heart failure	157	29.8	58	19.3	99	43.8	**< 0.001**
Coronary heart disease	95	18.1	41	13.7	54	23.9	**0.003**
Atrial fibrillation	101	19.2	37	12.3	64	28.3	**< 0.001**
Cardiovascular disease	216	41.1	83	27.7	133	58.8	**< 0.001**
COPD	51	9.7	21	7.0	30	13.3	**0.018**
Asthma	21	4.0	15	5.0	6	2.7	0.260
Obstructive airway disease	66	12.5	34	11.3	32	14.2	0.354
Lung fibrosis	9	1.7	6	2.0	3	1.3	0.739
Autoimmune disorder	24	4.6	13	4.3	11	4.9	0.834
Malignant disease	105	20	33	11.0	72	31.9	**< 0.001**
Renal disease	127	24.1	39	13.0	88	38.9	**< 0.001**
Hepatic disease	51	9.7	26	8.7	25	11.1	0.375
Cerebrovascular disease	86	16.3	34	11.3	51	22.6	**< 0.001**
Dementia	79	15.0	8	2.7	71	31.4	**< 0.001**
PAD	37	7.0	14	4.7	23	10.2	**0.016**
VTE	24	4.6	14	4.7	10	4.4	1.000
Depression/psychiatric disease	87	16.5	41	13.7	46	20.4	**0.044**
Other diseases	358	68.1	193	64.3	165	73.0	**0.038**

Bold p-values represent statistical significance (< 0.05)

Cardiovascular disease: At least one of the cardiac comorbidities (see text); COPD: Chronic obstructive pulmonary disease; Obstructive airway disease: At least one of the two comorbidities (see text); PAD: peripheral artery disease; VTE: venous thromboembolism

### Treatment limitations according to PTL

Among the 526 patients, 300 (57.0%) declared no therapy limitations, 32 (6.1%) one limitation, 23 (4.4%) two limitations, and 171 (32.5%) three limitations. Specifically, 175 patients (33.3%) refused transfer to ICU, 194 (36.9%) orotracheal intubation (DNI), and 222 (42.2%) cardiopulmonary resuscitation (DNR). There was a large overlap, as 172 patients refused both ICU and intubation, 171 both ICU and reanimation, and 193 both intubation and reanimation.

### Clinical predictors of treatment limitations

Age, diastolic blood pressure, body temperature, SpO_2_, major symptoms, comorbidities, hemoglobin, creatinine and CRP significantly differed between patients with no versus at least one treatment limitation (Tables 1, 2). The results of ROC analyses for the binary categories of laboratory parameters are given in Additional file 1 and illustrated in Additional file 2: Figure S1. In logistic regression analyses, the single limitations were linked to age (p = 0.001 each), dementia (p = 0.001 each) and eGFR < 55 ml/min (p < 0.05 each), but not sex, peripheral artery disease, obstructive airway disease and malignant disease. The CCI and cardiovascular disease were associated only with the refusal of resuscitation (p < 0.05 each). The results for a combined variable denoting the presence of at least one treatment limitation are shown in Table 3, confirming age, cardiovascular disease, dementia, CCI and eGFR as significant predictors.

**Table 3:** Predictors of the presence of at least one treatment limitation

Predictor	b	SE	OR	95% CI		p-value
Sex (female vs. male)	− 0.47	0.33	0.63	0.29	1.15	0.129
Age	0.13	0.02	1.14	1.11	1.20	**0.001**
PAD	0.32	0.79	1.37	0.28	7.54	0.664
Cardiovascular disease	− 0.73	0.37	0.48	0.23	0.95	**0.033**
Obstructive airway disease	0.32	0.47	1.38	0.58	3.42	0.482
Malignant disease	0.71	0.42	2.03	0.97	5.21	0.074
Dementia	2.06	1.16	7.87	3.35	33.5	**0.001**
CCI	0.17	0.09	1.19	1.02	1.45	**0.036**
eGFR ≤ 55 (ml/min)	0.95	0.32	2.57	1.43	5.00	**0.002**
SpO_2_ ≤ 93%	− 0.34	0.33	0.71	0.37	1.34	0.289
Neutrophils ≥ 5 (G/l)	0.11	0.33	1.11	0.58	2.08	0.723
CRP ≥ 7 (mg/dl)	− 0.04	0.33	0.96	0.49	1.82	0.894

For cardiovascular disease and obstructive airway disease compare with Table 2. The results shown are based on 439 patients having complete data for all of the predictors included

Bold p-values represent statistical significance (< 0.05)

*b* Regression coefficient, *SE* standard error of regression coefficient, *OR* odds ratio corresponding to the regression coefficient, *CI* confidence interval of odds ratio, *PAD* peripheral artery disease, *CCI* Charlson comorbidity index, *eGFR* estimated glomerular filtration rate

Using the probabilities derived from logistic regression in combination with ROC analyses to predict treatment limitations, the accuracy compared with the actual limitations was 82.0, 82.5 and 83.4% for ICU, intubation and cardiopulmonary resuscitation, respectively. The sensitivity for correctly predicting one of the limitations ranged between 84.0 and 91.5%, and specificity between 77.2 and 81.5%.

### Mortality and prognostic factors

Mortality was 27.2% in the total population, specifically 20.3% in patients not treated on ICU and 49.2% in ICU patients. In ICU patients without MV mortality was 30.8% and in ICU patients with MV (n = 74) 62.2%. The clinical characteristics of patients who survived versus those who died are given in Additional file 1: Table S1. Moreover, the distribution of deaths from COVID-19 over time is shown in Additional file 3: Figure S2, illustrating the rise of the epidemic until the midst of April 2020 and the subsequent rapid decline.

**Fig. 1:**
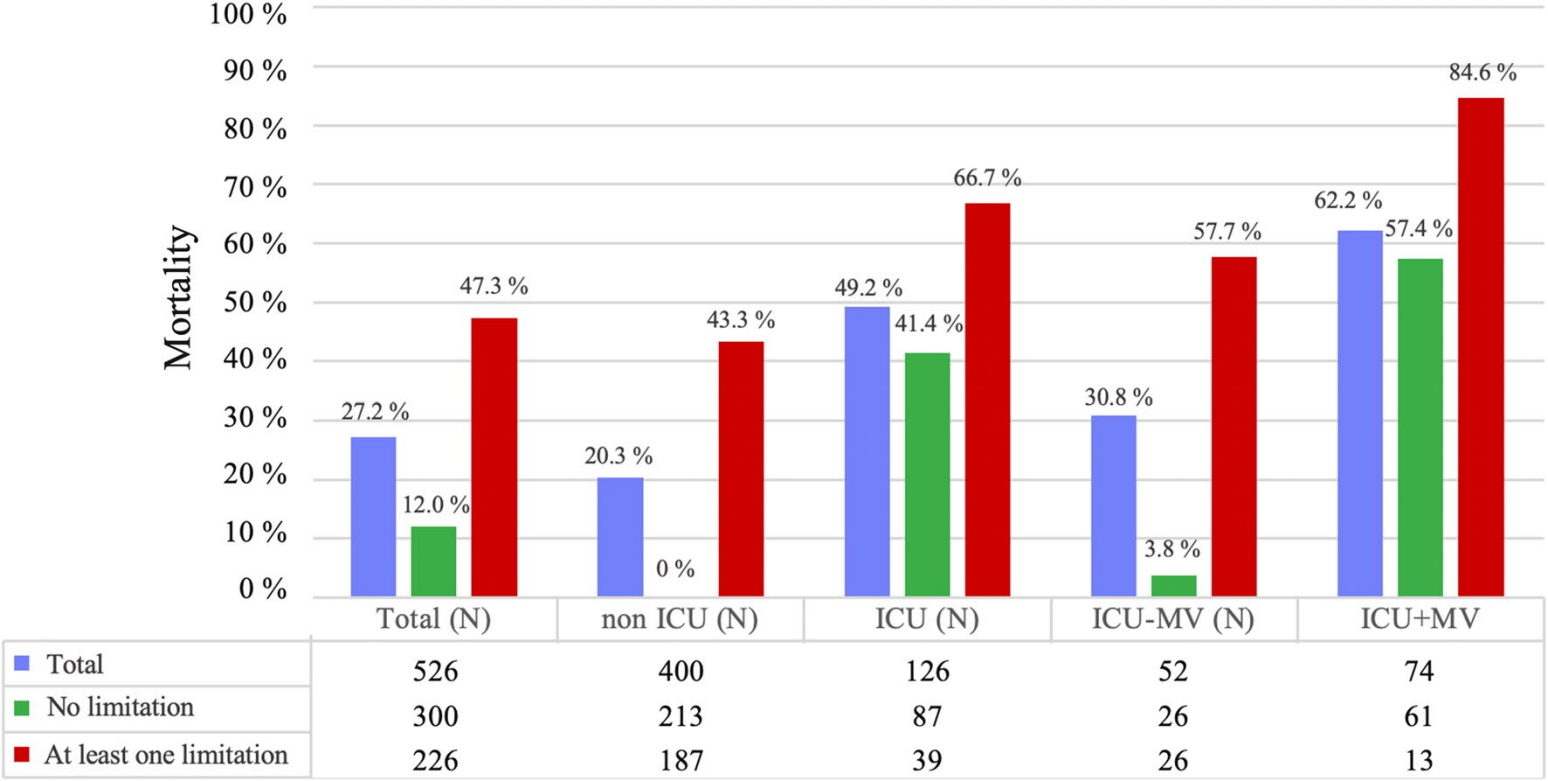
Mortality in the total population, non ICU and ICU patients as well as and ICU patients without or with mechanical ventilation (MV). The numbers given below the figure show the size of the group to which the percentages refer. *ICU* Intensive care unit, *MV* mechanical ventilation, *ICU + MV *ICU with mechanical ventilation, *ICU − MV* ICU without mechanical ventilation

We next analyzed mortality as a function of treatment limitations and the different treatment conditions regarding ICU and MV. When stratifying according the presence of at least one versus no limitation, those with at least one PTL showed an overall mortality of 47.3%, of 43.3% for non-ICU patients and of 66.7% for ICU patients, moreover of 57.7% in ICU patients without MV and of 84.6% in ICU patients with MV. Conversely, in patients without limitations, overall mortality was 12.0%, that of non-ICU patients 0.0% and of ICU patients 41.4%, and that of ICU patients without and with MV 3.8% and 57.4%, respectively. The percentages and numbers, to which the percentages refer, are given in Fig. 1.

First, mortality was statistically compared between different treatment conditions, either within the total group or within the two groups without and with any treatment limitation. In the total group, as well as in the groups without and with any treatment limitation, there were always significant differences in mortality between the non-ICU and ICU groups (p < 0.01 each). This was also true for the comparison of mortality between ICU without MV and ICU with MV, except for the group with any limitation (p = 0.151), probably due to low case numbers in this subgroup.

Second, mortality was statistically compared between the two groups without and with any treatment limitation for each treatment condition including ICU and MV. In the total group, and the non-ICU, ICU and ICU-non-MV subgroups, mortality was always higher in the group with any limitation compared to the group without limitation (p < 0.05 each). This was not true for the patients admitted to ICU and MV (p = 0.113), probably again due to low case numbers.

In the next step, independent predictors of death were identified by logistic regression analysis. When excluding PTL as predictors, the CCI, eGFR, neutrophil number, CRP and SpO_2_ turned out to be significant (p < 0.05 each), whereas single comorbidities were not statistically significant (Table 4). Additional analyses with forward and backward variable selection that were performed to detect predictors possibly masked by collinearity, confirmed these predictors as significant.

**Table 4:** Predictors of mortality without taking into account treatment limitations

Predictor	b	SE	OR	95% CI		p-value
Sex (female vs. male)	− 0.37	0.30	0.69	0.38	1.17	0.190
Age	0.03	0.02	1.03	1.00	1.06	0.054
PAD	0.41	0.56	1.51	0.50	4.57	0.409
Cardiovascular disease	− 0.48	0.37	0.62	0.29	1.27	0.177
Obstructive airway disease	− 0.15	0.43	0.86	0.34	1.90	0.712
Malignant disease	− 0.06	0.42	0.94	0.39	2.05	0.875
Dementia	0.63	0.39	1.87	0.91	4.26	0.088
CCI	0.24	0.09	1.27	1.09	1.58	**0.004**
eGFR ≤ 55 (ml/min)	0.92	0.31	2.50	1.34	5.00	**0.001**
SpO_2_ ≤ 93%	0.61	0.30	1.85	1.09	3.49	**0.029**
Neutrophils ≥ 5 (G/l)	0.63	0.31	1.88	1.01	3.49	**0.039**
CRP ≥ 7 (mg/dl)	1.61	0.33	4.88	2.94	10.59	**0.001**

For the definition of abbreviations see Tables 2, 3. The results shown are based on 439 patients having complete data for all of the predictors included

Bold p-values represent statistical significance (< 0.05)

**Fig. 2:**
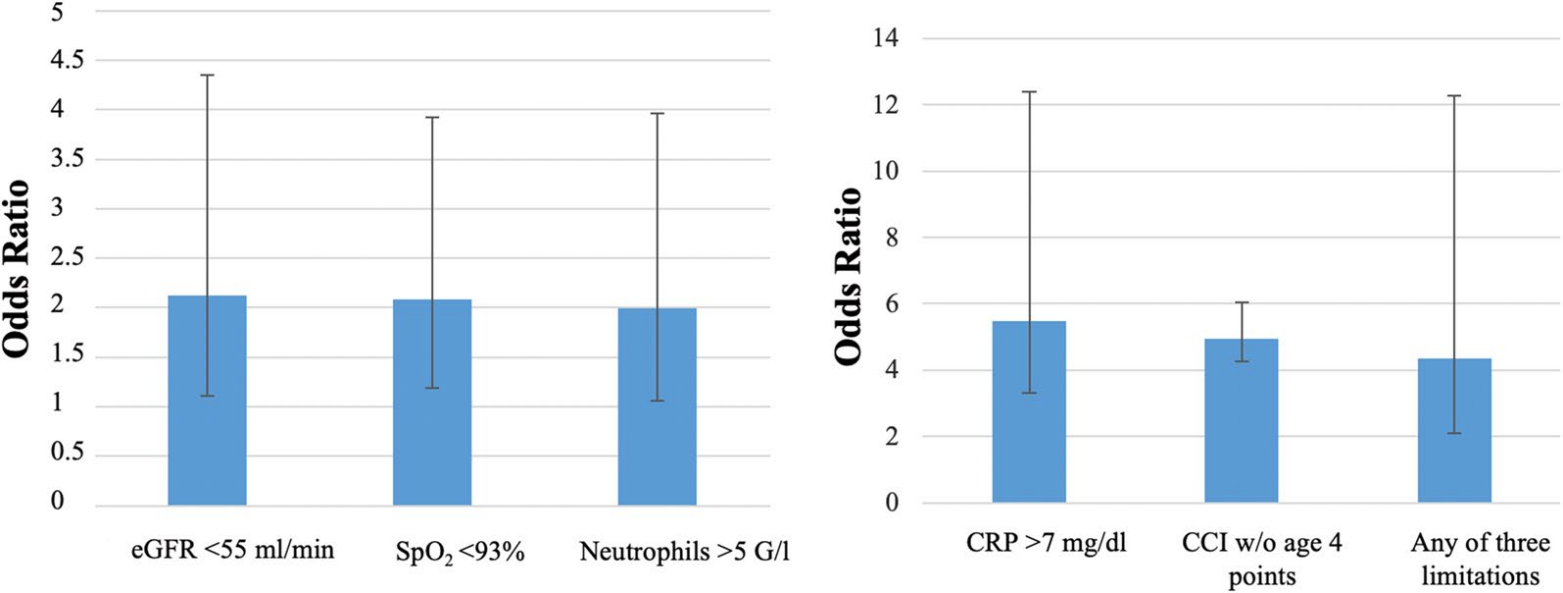
Odds ratios for predictors of mortality including treatment limitations based on the results of Table 5, and their 95% confidence intervals. Due to the difference in maximum values, two panels with different scales have been chosen. For the definition of abbreviations, see Table 3

In subsequent analyses, each of the three PTL limitations was added as a predictor. All PTL were significantly related to death (p = 0.001 each), whereby the CCI, eGFR, neutrophil number, CRP and SpO_2_ were always additional predictors (p < 0.05 each). To summarize the findings, we used the combined variable indicating the presence of at least one treatment limitation; the results are given in Table 5. The combined PTL was a highly significant predictor of death (p = 0.001), and the CCI remained significant, as well as eGFR, neutrophil number, CRP and SpO_2_ (p < 0.05 each). This result was robust when using either forward or backward stepwise selection of variables, which did neither remove nor add further predictors. Corresponding odds ratios are visualized in Fig. 2.

**Table 5:** Predictors of mortality including the presence of at least one treatment limitation

Predictor	b	SE	OR	95% CI		p-value
Sex (female vs. male)	− 0.43	0.32	0.65	0.33	1.17	0.161
Age	0.00	0.02	1.00	0.97	1.04	0.893
PAD	0.35	0.57	1.42	0.52	4.31	0.510
Cardiovascular disease	− 0.39	0.39	0.68	0.28	1.32	0.281
Obstructive airway disease	− 0.17	0.46	0.84	0.30	1.99	0.681
Malignant disease	− 0.27	0.44	0.76	0.30	1.65	0.531
Dementia	0.27	0.43	1.31	0.55	2.97	0.508
CCI	0.21	0.09	1.24	1.07	1.51	**0.010**
eGFR ≤ 55 (ml/min)	0.75	0.35	2.11	1.12	4.35	**0.020**
SpO_2_ ≤ 93%	0.73	0.31	2.08	1.20	3.94	**0.012**
Neutrophils ≥ 5 (G/l)	0.69	0.33	1.99	1.06	3.97	**0.027**
CRP ≥ 7 (mg/dl)	1.70	0.34	5.49	3.32	12.43	**0.001**
Limitation (at least one)	1.47	0.45	4.34	2.10	12.30	**0.001**

For the definition of abbreviations see Tables 2, 3. The results shown are based on 439 patients having complete data for all of the predictors included

Bold p-values represent statistical significance (< 0.05)

## Discussion

The present study was based on data from a region with high incidence of SARS-CoV-2 infection during the initial wave of COVID-19. To the best of our knowledge, it comprises the largest German cohort of COVID-19 patients with detailed and comprehensive clinical data in individual patients. The study demonstrated the great importance of patients’ treatment limitations (PTL) for mortality from this disease. Between 33 and 42% of patients had at least one limitation regarding rejection of admission to ICU, or intubation, or cardiopulmonary resuscitation. Limitations were associated with age, dementia, the CCI and renal function in terms of eGFR. Their association with mortality was strong and robust, and independent from other predictors such as the burden from comorbidities, oxygen saturation by pulse oximetry, renal function, CRP and neutrophil number upon hospital admission. In summary, PTL appeared as a further, comprehensive, previously unrecognized determinant of death from COVID-19 that added to or partially replaced known predictors. This novel finding might be useful for a more detailed understanding of the mortality from COVID-19 including the large variations across countries and locations.

With regard to age and sex, the present population was similar to cohorts of SARS-CoV-2 patients from Germany [3, 20] showing a median age of 72 and 73 years, respectively, and a slightly higher percentage of males (51.8 and 51.5%, respectively). In line with these studies or findings in other European regions and countries [4, 5, 10, 11], we observed that most patients had a least one major comorbidity. Among these, systemic hypertension, left-heart failure, renal disease, diabetes and malignant disease were most frequent. Interestingly, in the study by Nachtigall and co-workers [3] the proportion of patients with at least one comorbidity was only about half as large as in our cohort. Differences in comorbidities between studies were also reflected in the CCI. When computed without age, about 53% of patients of our cohort showed a CCI of ≥ 2, and when including age, this percentage raised to about 79%, compared to 55% in the study by Karagiannidis and co-workers [20]. The relevance of comorbidities and age and their differences between populations was also evident for treatment limitations. When not taking into account age, the median CCI was 1 in patients without limitations and 3 in patients with at least one limitation but when computing the CCI with age, the respective median values were 1 and 7.

In the total population of hospitalized patients, mortality was 27.2%, similar to that of the prospective observational UK cohort study [4] but higher than in German cohorts (22% and 16.6%) [3, 20], although the distributions of age and sex in our study and the previous two studies from Germany were not much different [3, 20]. In the PRECOVID study from Spain [5], 771 of 3641 patients (21%) died irrespective of hospitalization. Thus, mortality was in the upper range in our cohort, and this might have been related to the large frequency of treatment limitations as underlined by Fig. 1.

An important recent observation was that in patients admitted to hospital with COVID-19, outcomes were better predicted by frailty than by age or comorbidity [21]. These factors are closely related to treatment limitations that might affect mortality risk by limiting the scope of interventions. Such limitations are well known as DNR and DNI statements regarding resuscitation and intubation, respectively. We added the acceptance of ICU treatment as a third, more general limitation that turned out to be informative. It appears surprising that these limitations have not been explicitly addressed in COVID-19 patients, even more, as we found the proportion of patients with treatment limitations to be one third and more. Clinical experience shows that decisions on treatment limitations are founded in objective medical factors including age and comorbidities [22], but also personal attitudes and preferences, both on the patients’ or relatives’ and the physicians’ side. The complex interplay between these factors is reflected in changes occurring after consultation and discussion [22], and it is probably impossible to disentangle these factors retrospectively in detail. Irrespective of this, major objective determinants could be identified in our study (Tables 1, 2 and 3). In patients with treatment limitations, mortality rate reached 85% in the subgroup of ICU-patients with mechanical ventilation. This high mortality corresponds to the high rate of DNR in this population. It might also be related to a higher use of opioids as previously reported in patients with DNI/DNR limitations [23]. In contrast, overall mortality rate appeared low (12%) in patients without any treatment limitation. Future studies might show whether these limitations explain part of the variation of mortality from COVID-19 within and across countries.

As independent predictors of treatment limitations, we found age, dementia, cardiovascular disease, CCI and eGFR being < 55 ml/min, a result which appears plausible from both the patients’ and the physicians’ perspective. Regarding the accuracy of prediction, the contribution of these objective measures to the final PTL was 80% and more. The remaining 20% obviously comprised other factors including subjective factors. In line with proposals made previously [17], this observation could be helpful in understanding decision making in a severe life-threatening disease such as COVID-19. The most remarkable finding was that treatment limitations were related to mortality beyond known prognostic factors by integrating some though not all of these into a comprehensive indicator (see Fig. 2). This suggests that they constitute an individual factor having considerable impact on the prognosis in COVID-19. Noteworthy enough, each of the three PTL items was a significant predictor (p < 0.001 each) associated with an increase in mortality risk by a factor of about 4 (data not shown). Due to their close connection we considered their pooling into a single variable as justified, however, when ranking their importance in a stepwise variable selection, DNR turned out to be the dominant predictor.

In line with the literature, we found age, blood pressure, SpO_2_, comorbidities, hemoglobin, leucocyte, lymphocyte and neutrophil numbers, creatinine, eGFR, LDH, CRP and IL-6 to be predictive for mortality [5, 10–12, 16, 24, 25]. ROC analyses (see Additional file 2: Figure S1) yielded cut-off values regarding neutrophil number ≥ 5 G/l, CRP level > 7 mg/l, eGFR < 55 ml/min and SpO_2_ < 93% which were identified as independent risk factors for mortality in multivariate analyses. Noteworthy enough, the predictive value of eGFR upon admission was superior to that of the corresponding creatinine value or the diagnosis of a preexisting renal disease. The deleterious effect of renal impairment is probably linked to endothelial dysfunction and increased cardiovascular risk, both of which affect the outcome of COVID-19 [26]. In the current analysis, eGFR appeared of particular interest, as it was a strong predictor of both, treatment limitations and mortality.

### Limitations

The present analysis comprised only a limited number of patients with SARS-CoV-2 infection compared to large studies reported in the literature [4, 5, 10, 20, 27]. On the other hand, we performed a comprehensive, standardized assessment of patients allowing the evaluation of clinical management and risk factors in a hotspot region. We restricted our analysis to the initial wave of COVID-19 with its sharp rise and decline (see Additional file 3: Figure S2), as this provided high case numbers and fairly homogeneous conditions. The prognostic values of markers such cardiac troponin [16] and d-dimer [25, 28] could not be evaluated in the total population, because the respective kits differed between locations and were difficult to compare; when analyzing the Rosenheim data alone, troponin was significantly related to mortality, as expected (data not shown). Furthermore, detailed information on body mass index (BMI) and smoking status, which have been identified as additional prognostic markers in large cohorts with COVID-19 [4, 29, 30], was not consistently available from the files. Moreover, we did not have detailed information on the processes by which treatment limitations were determined in each single case, and relied on the well-established, routine procedure involving patients, relatives and treating physicians.

## Conclusion

In a German hot-spot region of SARS-CoV-2 infections, in-hospital mortality was high, especially in patients with mechanical ventilation. It was considerably elevated in patients with treatment limitations that were present in a high number of patients. Treatment limitations were linked to age, comorbidity burden as summarized in the CCI, dementia, cardiac disease and reduced eGFR. They were a strong, independent factor in predicting mortality, in addition to reductions in eGFR and oxygen saturation and increases in neutrophil number and CRP levels assessed upon hospital admission. Based on these findings, patients’ individual treatment limitations appear to be an important factor for the outcome in COVID-19 and are probably worth to be taken into account in future studies, as they might explain part of the variation within and across countries in this pandemic.

## Supplementary Information

**Additional file 1.** Receiver operating characteristics analyses of independent predictors of mortality.

**Additional file 3: Figure S1.** Results of ROC analyses for eGFR, SpO_2_, CRP and Neutrophil number. Definition of abbreviations: ROC = Receiver Operating Characteristics, eGFR = estimated glomerular filtration rate; CRP = C-reactive protein; SpO_2_ = oxygen saturation from pulse oximetry.

**Additional file 3: Figure S2.** Distribution of deaths from COVID-19 over time. The bars show the numbers of deaths within each bin.

## Data Availability

The basic data are part of the Covid-DB. They are available after agreement by all authors of the present work for defined study purposes.

## References

[1] Zhu N, Zhang D, Wang W, Li X, Yang B, Song J, Zhao X, Huang B, Shi W, Lu R (2020). A novel coronavirus from patients with pneumonia in China, 2019. N Engl J Med.

[2] University JH. https://coronavirus.jhu.edu/map.html. Accessed 15 Oct 2020.

[3] Nachtigall I, Lenga P, Jozwiak K, Thurmann P, Meier-Hellmann A, Kuhlen R, Brederlau J, Bauer T, Tebbenjohanns J, Schwegmann K (2020). Clinical course and factors associated with outcomes among 1904 patients hospitalized with COVID-19 in Germany: an observational study. Clin Microbiol Infect.

[4] Docherty AB, Harrison EM, Green CA, Hardwick HE, Pius R, Norman L, Holden KA, Read JM, Dondelinger F, Carson G (2020). Features of 20 133 UK patients in hospital with covid-19 using the ISARIC WHO Clinical Characterisation Protocol: prospective observational cohort study. BMJ.

[5] Poblador-Plou B, Carmona-Pirez J, Ioakeim-Skoufa I, Poncel-Falco A, Bliek-Bueno K, Cano-Del Pozo M, Gimeno-Feliu LA, Gonzalez-Rubio F, Aza-Pascual-Salcedo M, Bandres-Liso AC (2020). Baseline chronic comorbidity and mortality in laboratory-confirmed COVID-19 cases: results from the PRECOVID study in Spain. Int J Environ Res Public Health.

[6] Salje H, Tran Kiem C, Lefrancq N, Courtejoie N, Bosetti P, Paireau J, Andronico A, Hozé N, Richet J, Dubost C-L (2020). Estimating the burden of SARS-CoV-2 in France. Science.

[7] Robert-Koch-Institut. https://www.rki.de/DE/Content/InfAZ/N/Neuartiges_Coronavirus/Fallzahlen.html. Accessed 15 Oct 2020.

[8] Cheng B, Hu J, Zuo X, Chen J, Li X, Chen Y, Yang G, Shi X, Deng A (2020). Predictors of progression from moderate to severe coronavirus disease 2019: a retrospective cohort. Clin Microbiol Infect.

[9] Gao L, Jiang D, Wen XS, Cheng XC, Sun M, He B, You LN, Lei P, Tan XW, Qin S (2020). Prognostic value of NT-proBNP in patients with severe COVID-19. Respir Res.

[10] Grasselli G, Zangrillo A, Zanella A, Antonelli M, Cabrini L, Castelli A, Cereda D, Coluccello A, Foti G, Fumagalli R (2020). Baseline characteristics and outcomes of 1591 patients infected with SARS-CoV-2 admitted to ICUs of the Lombardy Region, Italy. JAMA.

[11] Guan WJ, Liang WH, Zhao Y, Liang HR, Chen ZS, Li YM, Liu XQ, Chen RC, Tang CL, Wang T (2020). Comorbidity and its impact on 1590 patients with COVID-19 in China: a nationwide analysis. Eur Respir J.

[12] Wendel Garcia PD, Fumeaux T, Guerci P, Heuberger DM, Montomoli J, Roche-Campo F, Schuepbach RA, Hilty MP, Investigators R-I (2020). Prognostic factors associated with mortality risk and disease progression in 639 critically ill patients with COVID-19 in Europe: initial report of the international RISC-19-ICU prospective observational cohort. EClinicalMedicine.

[13] Xu J, Yang X, Yang L, Zou X, Wang Y, Wu Y, Zhou T, Yuan Y, Qi H, Fu S (2020). Clinical course and predictors of 60-day mortality in 239 critically ill patients with COVID-19: a multicenter retrospective study from Wuhan, China. Crit Care.

[14] Pujadas E, Chaudhry F, McBride R, Richter F, Zhao S, Wajnberg A, Nadkarni G, Glicksberg BS, Houldsworth J, Cordon-Cardo C (2020). SARS-CoV-2 viral load predicts COVID-19 mortality. Lancet Respir Med.

[15] Chen R, Liang W, Jiang M, Guan W, Zhan C, Wang T, Tang C, Sang L, Liu J, Ni Z (2020). Risk factors of fatal outcome in hospitalized subjects with coronavirus disease 2019 from a nationwide analysis in China. Chest.

[16] Du RH, Liang LR, Yang CQ, Wang W, Cao TZ, Li M, Guo GY, Du J, Zheng CL, Zhu Q (2020). Predictors of mortality for patients with COVID-19 pneumonia caused by SARS-CoV-2: a prospective cohort study. Eur Respir J.

[17] Curtis JR, Kross EK, Stapleton RD (2020). The importance of addressing advance care planning and decisions about do-not-resuscitate orders during novel coronavirus 2019 (COVID-19). JAMA.

[18] Levey AS, Stevens LA, Schmid CH, Zhang YL, Castro AF, Feldman HI, Kusek JW, Eggers P, Van Lente F, Greene T, Coresh J (2009). A new equation to estimate glomerular filtration rate. Ann Intern Med.

[19] Charlson ME, Pompei P, Ales KL, MacKenzie CR (1987). A new method of classifying prognostic comorbidity in longitudinal studies: development and validation. J Chronic Dis.

[20] Karagiannidis C, Mostert C, Hentschker C, Voshaar T, Malzahn J, Schillinger G, Klauber J, Janssens U, Marx G, Weber-Carstens S (2020). Case characteristics, resource use, and outcomes of 10 021 patients with COVID-19 admitted to 920 German hospitals: an observational study. Lancet Respir Med.

[21] Hewitt J, Carter B, Vilches-Moraga A, Quinn TJ, Braude P, Verduri A, Pearce L, Stechman M, Short R, Price A (2020). The effect of frailty on survival in patients with COVID-19 (COPE): a multicentre, European, observational cohort study. Lancet Public Health.

[22] Lee S, Kim T, Lee E, Lee C, Kim H, Rhee H, Park SY, Son H-J, Yu S, Park JW (2020). Clinical course and molecular viral shedding among asymptomatic and symptomatic patients with SARS-CoV-2 infection in a community treatment center in the Republic of Korea. JAMA Internal Med.

[23] Stream S, Nolan A, Kwon S, Constable C (2018). Factors associated with combined do-not-resuscitate and do-not-intubate orders: a retrospective chart review at an urban tertiary care center. Resuscitation.

[24] Liu Y, Du X, Chen J, Jin Y, Peng L, Wang HHX, Luo M, Chen L, Zhao Y (2020). Neutrophil-to-lymphocyte ratio as an independent risk factor for mortality in hospitalized patients with COVID-19. J Infect.

[25] Yao Y, Cao J, Wang Q, Shi Q, Liu K, Luo Z, Chen X, Chen S, Yu K, Huang Z, Hu B (2020). D-dimer as a biomarker for disease severity and mortality in COVID-19 patients: a case control study. J Intensive Care.

[26] Cantaluppi V, Guglielmetti G, Dellepiane S, Marengo M, Mehta R, Ronco C (2020). A call to action to evaluate renal functional reserve in COVID-19 patients. Am J Physiol Renal Physiol.

[27] Cummings MJ, Baldwin MR, Abrams D, Jacobson SD, Meyer BJ, Balough EM, Aaron JG, Claassen J, Rabbani LE, Hastie J (2020). Epidemiology, clinical course, and outcomes of critically ill adults with COVID-19 in New York City: a prospective cohort study. Lancet.

[28] Ye W, Chen G, Li X, Lan X, Ji C, Hou M, Zhang D, Zeng G, Wang Y, Xu C (2020). Dynamic changes of D-dimer and neutrophil-lymphocyte count ratio as prognostic biomarkers in COVID-19. Respir Res.

[29] Ioannou GN, Locke E, Green P, Berry K, O’Hare AM, Shah JA, Crothers K, Eastment MC, Dominitz JA, Fan VS (2020). Risk factors for hospitalization, mechanical ventilation, or death among 10 131 US Veterans with SARS-CoV-2 infection. JAMA Netw Open.

[30] Williamson EJ, Walker AJ, Bhaskaran K, Bacon S, Bates C, Morton CE, Curtis HJ, Mehrkar A, Evans D, Inglesby P (2020). Factors associated with COVID-19-related death using OpenSAFELY. Nature.

