# Supplementary material for: Patients’ treatment limitations as predictive factor for mortality in COVID-19: results from hospitalized patients of a hotspot region for SARS-CoV-2 infections
Source: Respir Res. 2021 Jun 4;22:168. doi: 10.1186/s12931-021-01756-2 (PMC8182347; doi:10.1186/s12931-021-01756-2)
Supplement: Supplementary file 1 — Additional file 1. Receiver operating characteristics analyses of independent predictors of mortality. [file 12931_2021_1756_MOESM1_ESM.pdf]

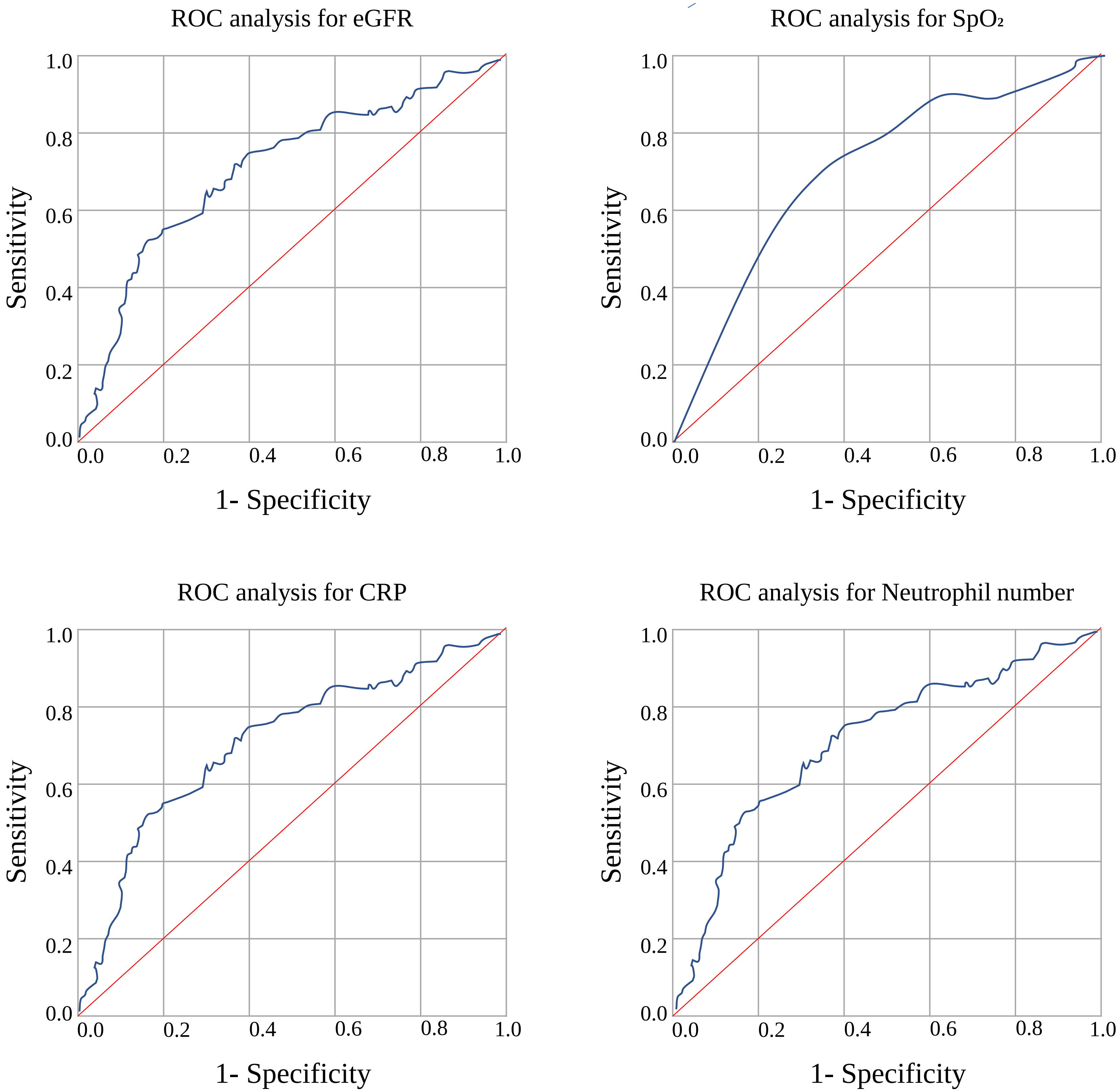
ROC analysis for eGFR
ROC analysis for SpO₂
ROC analysis for CRP
ROC analysis for Neutrophil number
Sensitivity
1- Specificity
0.0
0.2
0.4
0.6
0.8
1.0
