# Supplementary figures and images for: Patients’ treatment limitations as predictive factor for mortality in COVID-19: results from hospitalized patients of a hotspot region for SARS-CoV-2 infections
Source: Respir Res. 2021 Jun 4;22:168. doi: 10.1186/s12931-021-01756-2 (PMC8182347; doi:10.1186/s12931-021-01756-2)

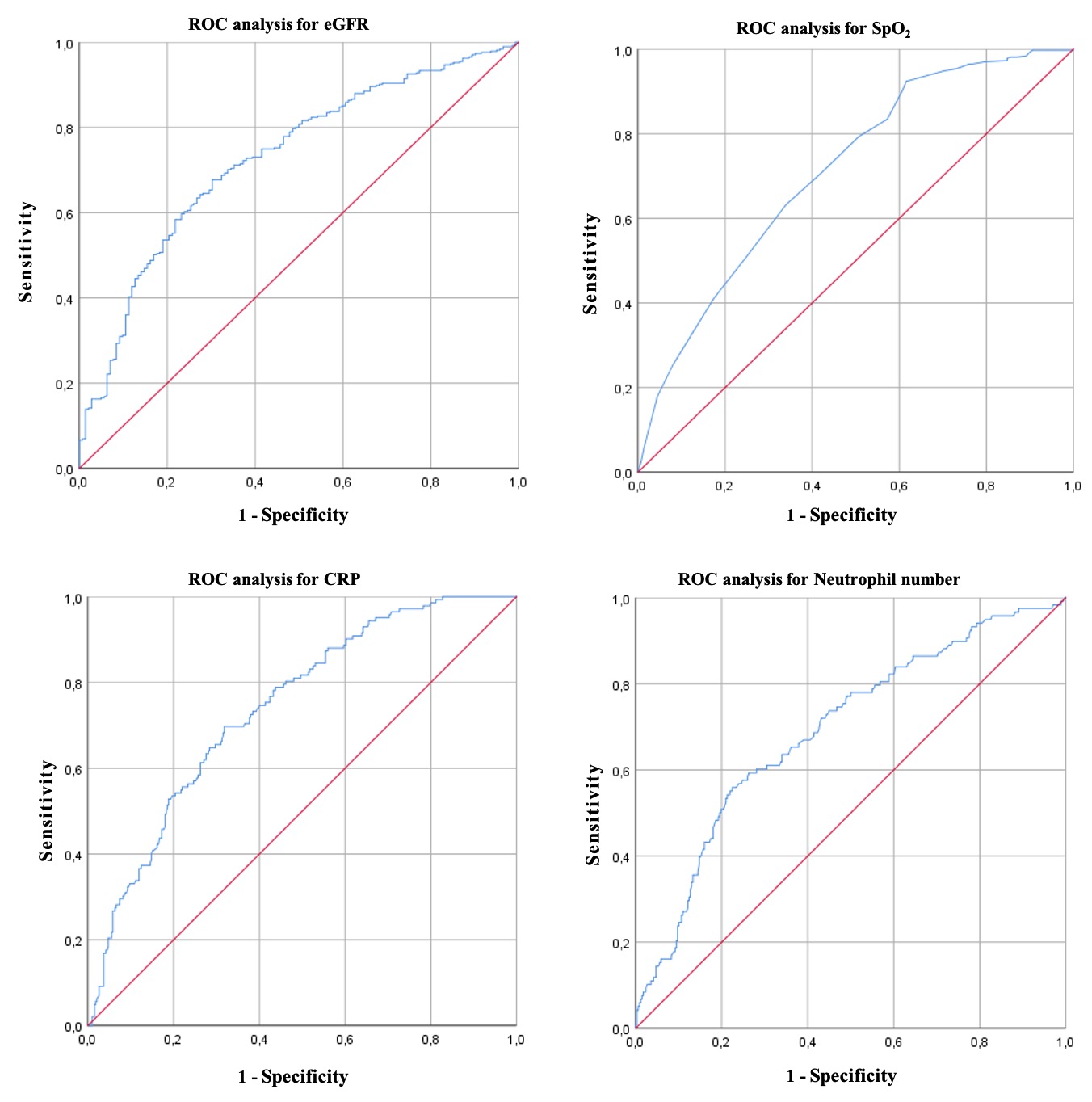

Supplement: Supplementary file 2 — Additional file 3: Figure S1. Results of ROC analyses for eGFR, SpO2, CRP and Neutrophil number. Definition of abbreviations: ROC = Receiver Operating Characteristics, eGFR = estimated glomerular filtration rate; CRP = C-reactive protein; SpO2 = oxygen saturation from pulse oximetry. [file 12931_2021_1756_MOESM2_ESM.jpg]

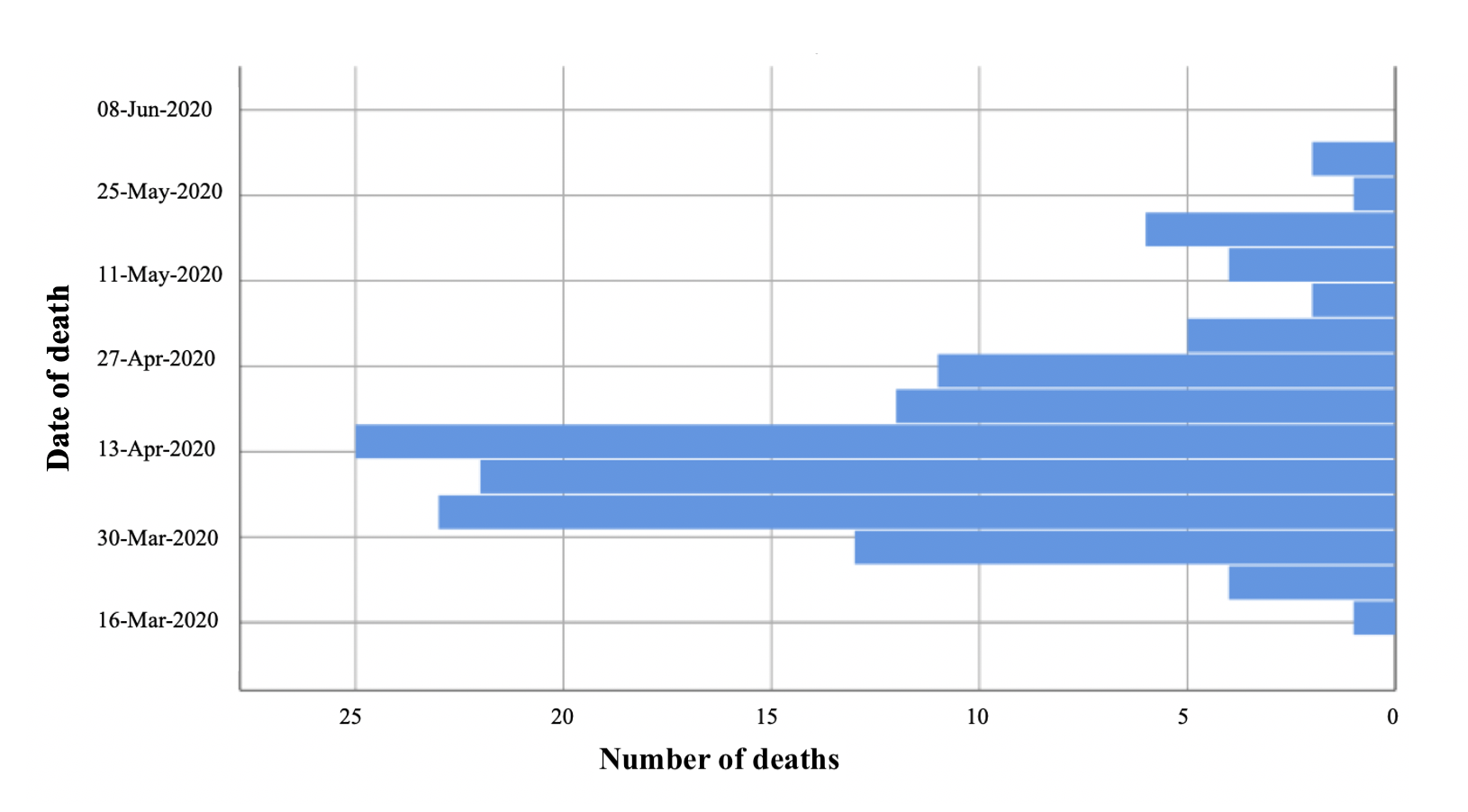

Supplement: Supplementary file 3 — Additional file 3: Figure S2. Distribution of deaths from COVID-19 over time. The bars show the numbers of deaths within each bin. [file 12931_2021_1756_MOESM3_ESM.png]
